# Co-encapsulation of temozolomide and PbS quantum dots in apoferritin for transferrin receptor 1 targeting, imaging and treatment of glioblastoma

**DOI:** 10.1039/d5na00557d

**Published:** 2025-10-03

**Authors:** Reyhan Dilsu Colpan, Ellie B. Ward, Dongling Zhang, Binbing Ling, Umar Iqbal, Maria Moreno, Neil R. Thomas, Lyudmila Turyanska, Tracey D. Bradshaw

**Affiliations:** a Biodiscovery Institute, School of Pharmacy, University of Nottingham NG7 2RD UK Tracey.Bradshaw@nottingham.ac.uk; b Faculty of Engineering, University of Nottingham Nottingham NG7 2RD UK Lyudmila.Turyanska@nottingham.ac.uk; c Biodiscovery Institute, School of Chemistry, University of Nottingham NG7 2RD UK; d National Research Council Canada Ottawa Canada

## Abstract

Apoferritin (AFt) nanocages represent a promising drug delivery platform by targeting transferrin receptor 1 (TfR1) that is abundantly expressed on both blood–brain barrier (BBB) endothelial and glioma cells, offering promising opportunities for intractable brain cancer treatment. We report the development of a theranostic agent based on lead sulfide quantum dots (PbS QDs) and temozolomide (TMZ) co-encapsulated inside horse spleen AFt cages (AFt-PbS-TMZ). *In vitro* evaluation of AFt-PbS-TMZ revealed cancer-selective enhancement of growth inhibition in glioblastoma (GBM) cells (U373M, U373V, and U87MG) compared to non-encapsulated agents. These findings in two-dimensional cultures were further corroborated by the results in three-dimensional 3D U87MG tumour spheroids, where the use of AFt-PbS-TMZ significantly enhanced TMZ efficacy; the treatment resulted in a significant (*p* < 0.0001) decrease in spheroid volumes and cell viability. Additionally, the near-infrared emission of the PbS QDs enabled imaging of the nanoparticle delivery. The emission of the PbS QDs was clearly detectable within the cell spheroids, even at concentrations significantly lower than GI_50_ values, offering opportunities for non-invasive deep tissue imaging. These results reveal that AFt-PbS-TMZ can be an efficient theranostic agent for targeted cancer drug delivery, addressing limitations associated with current treatment and therapeutic monitoring.

## Abstract 2

Co-encapsulation of PbS quantum dots and temozolomide within apoferritin enables enhanced cancer-selective therapeutic and imaging capability in glioblastoma cells.

## Graphical abstract

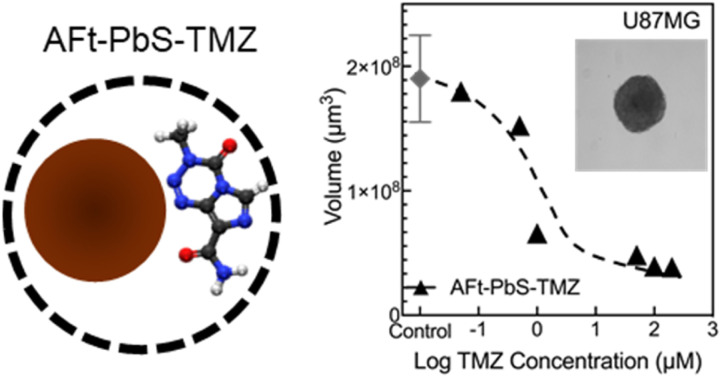

## 1 Introduction

Glioblastoma multiforme (GBM) is an aggressive brain tumour with high recurrence (∼90%), high mortality, (>90%), and low cure rates.^1^ Temozolomide (TMZ) is a standard-of-care oral DNA-alkylating drug, with the mechanism of action relying of methylating *O*6-guanine, arresting the GBM cell cycle at the G2/M phase, producing DNA double-strand breaks, and leading to apoptotic or autophagic death of GBM cells.^2^ The ability of TMZ to cross the blood–brain barrier (BBB), due to its small size and lipophilic nature (clog *P* = 0.81),^3^ is directly linked to its efficacy in treating brain cancers.^4,5^ However, non-specific toxicity, drug resistance, and poor drug accumulation at the tumour site limit treatment success.^1^ TMZ drug resistance is often associated with overexpression of *O*6-methylguanine-DNA methyltransferase (MGMT), deficiency in DNA mismatch repair (MMR), and the presence of drug efflux transporters on BBB endothelia including permeability glycoprotein (P-glycoprotein; P-gp).^6,7^ To improve GBM management, TMZ formulations have been developed using drug delivery systems, such as protein,^7^ lipid,^8^ and polymeric^9^ nanocarriers. These formulations have been functionalized with peptides,^10^ transporters, and transferrin or receptors^11,12^ to specifically target tumour cells or the BBB.^2,13^ Of particular interest is the development of theranostic approaches, where imaging and therapeutic capabilities are combined within one delivery vehicle to achieve improved tumour detection, optimize dosing regimens, and enable and monitor innovative treatment strategies.^14^

Semiconductor quantum dots (QDs) have emerged as promising imaging agents,^15^ due to their tunable optical properties, high quantum yield and resistance to photobleaching,^15–17^ QDs, such as PbS, with photoluminescence in the near infrared region (NIR-II, 1000–1700 nm), where biological tissue absorption, autofluorescence and light scattering are low, offer significant advantages for non-invasive deep tissue imaging.^18^ Nanoscale drug delivery systems have been designed to combine imaging and therapeutic agents within a single construct, as demonstrated by the development of antibody-functionalized liposomes with encapsulated docetaxel and CdSe/ZnS QDs.^19^ Furthermore, these systems offer additional opportunities for specific targeting, *e.g.* integrating or incorporating targeting agents that facilitate transcytosis across the BBB.^2^

Among nanoscale carriers, the protein apoferritin (AFt) has gained considerable attention, as it is biocompatible and biodegradable, and has a nanoscale size (∼12 nm external and 8 nm internal cavity diameters).^20^ By exploiting the overexpression of transferrin receptor 1 (TfR1) on cancer and BBB endothelial cells,^7,21^ AFt can cross the BBB by transcytosis and selectively target cancer cells,^22^ offering potential solutions to the limitations of conventional TMZ treatment and GBM diagnostic methods. AFt has been successfully employed to encapsulate individual cargo, such as anti-cancer drugs (TMZ, doxorubicin (DOX), and gefitinib^7,23,24^) and nanoparticles (AuNPs^25^ and QDs^26^). As such, AFt holds significant promise in cancer theranostics;^27^ however, to date, there have been only a few attempts to encapsulate combined agents within one cage; examples include curcumin and gadolinium (Gd),^28^ as well as graphene QDs, iron oxide and DOX.^29^ Hence there is a significant need for development of robust and optimised processes for co-encapsulation of multiple agents within one AFt nanocarrier, with particular potential benefit in GBM treatments. To enhance the translation of findings from *ex vivo* to *in vivo* studies, three-dimensional (3D) cultures have emerged as a superior model compared to traditional two-dimensional (2D) cultures, as they more accurately recapitulate the tumour microenvironment by preserving critical features such as cell–cell interactions and extracellular matrix (ECM) composition, which are essential for *in vivo* relevance.^30^ Despite significant interest, development of co-encapsulation of multifunctional agents remains challenging, and combination of imaging and therapeutic agents within a nanoscale vehicle capable of targeting cancer cells that over-express receptors, as well as penetrate the BBB, is of particular interest for identifying the next generation of therapies for brain cancers.

In this study, we report the development of a theranostic agent, AFt-PbS-TMZ, in which PbS QDs provide imaging capability in the near-infrared wavelength range of low biological tissue absorption, TMZ serves as a treatment agent and the delivery vehicle, AFt, is capable of crossing the blood brain barrier and targetting glioblastoma cells. To form AFt-PbS-TMZ, a two-step co-encapsulation protocol was established, in which reassembly of the AFt capsule is used to entrap PbS QDs within the AFt cavity, followed by in-diffusion of TMZ through the 3- and 4-fold channels of the AFt capsule. The process was optimized to ensure that the structural integrity and exterior surface properties of the protein capsule remained intact, hence retaining its TfR1-targeting ability. The activity of this formulation was assessed *in vitro* in 2D cultures, as well as in 3D cultures, which represent a more accurate platform for evaluation of AFt-based theranostics, particularly in challenging malignancies such as GBM. Growth inhibitory studies were conducted with AFt, PbS QDs, TMZ, AFt-PbS, AFt-TMZ, and AFt-PbS-TMZ in U373M, U373V and U87MG GBM tumour cell lines, as well as in non-tumour human astrocytes and MRC-5 fibroblasts. Additionally, U87MG tumour spheroids were used to further elucidate therapeutic activity. These studies, in combination with NIR optical imaging confirmed the theranostic potential of the AFt-PbS-TMZ formulation.

## 2 Materials and methods

### 2.1 Materials

Horse spleen AFt was obtained by chemical removal of Fe from horse spleen ferritin (Sigma Aldrich). TMZ, DMSO and all reagents used for PbS QD synthesis were purchased from Sigma Aldrich, unless otherwise noted. T25/T75 flasks (Corning), 96 well plates (ThermoFisher Scientific), ultra-low attachment (ULA) plates (ThermoFisher) were used for *in vitro* cell culture studies. MTT was purchased from Sigma Aldrich; PB cell viability reagent was purchased from ThermoFisher Scientific (CAT:A13262). Recombinant anti-human TfR1 monoclonal antibodies (CAT:136800) and goat anti-mouse IgG (H + L) superclonal secondary antibodies (CAT:A28177) were purchased from ThermoFisher, and anti-human GAPDH monoclonal antibodies (CAT:G8795) were obtained from Sigma Aldrich. A 4% paraformaldehyde solution (ThermoFisher), Grace Biolabs (Merck, GBL654002) and mounting medium fluoromount G (CliniciSciences) were used for 3D spheroid imaging.

### 2.2 PbS QD and TMZ encapsulation into horse spleen apoferritin

For the co-encapsulation of PbS QDs and TMZ, pH-dependent disassembly and passive diffusion methods were used. AFt was produced by reductive dissolution of the iron core of horse spleen ferritin (Sigma Aldrich,^31^). PbS QDs were synthesized according to the published method of Hennequin *et al.*^32^ AFt-PbS samples were produced by mixing 1 : 1 (v/v%) ratio of PbS QDs (5 mg mL^−1^) and disassembled AFt (pH = 2) (3 mg mL^−1^). The solution was purified using an Amicon ultra 4 mL centrifuge filter (30 kDa molecular weight cutoff (MWCO), Merck Millipore, USA), and subsequently dialyzed against 20 mM HEPES buffer (pH 7.4, room temperature, N_2_ flow) for 2 days. TMZ was dissolved in DMSO (10 mM, 7.2 μmol) and added dropwise every 30 minutes (min) to the AFt-PbS solution (2 mL) under stirring at 4 °C to achieve a final encapsulation ratio of 1 : 400 TMZ per AFt capsule. The resulting solution was centrifuged (4000*g*, 4 min) to remove unencapsulated TMZ and filtered through a 0.22 μm syringe filter. The final AFt-PbS-TMZ solutions were stored at 4 °C under a N_2_ atmosphere.

### 2.3 Dynamic light scattering (DLS) studies

A Malvern Zetasizer Nano ZS instrument was used to measure hydrodynamic size and zeta-potential. The samples were diluted in 0.1 M NaOAc buffer (pH 5.5) or in Millipore water to a concentration of 0.5 mg mL^−1^ and were filtered using a 0.22 μm syringe filter. All measurements were performed at room temperature (20 °C) in triplicate.

### 2.4 Photoluminescence (PL) studies

The photoluminescence (PL) spectra were recorded using a Horyba LabRam system equipped with an InGaAs detector. Excitation was provided by a HeNe laser (*λ* = 633 nm).

### 2.5 Near-infrared imaging

The IMA™ (Photonetc, Montreal, Canada) imaging system was used to evaluate the deep tissue imaging capabilities of PbS QDs. Short wave infrared (SWIR) wavelength imaging was performed using a 785 nm laser at 1.5 W or 20% power, a 980 nm or a 1000 nm long-pass (LP) emission filter, and a camera exposure time of 0.03 or 0.1 seconds (s). PbS QDs′ deep tissue imaging capabilities were investigated in GBM cell spheroids after treatment with PbS QDs and AFt-PbS-TMZ. On day 7, post treatment with PbS QDs and AFt-PbS-TMZ (50 μg mL^−1^), spheroids were washed with phosphate buffer saline (PBS) solution and fixed with 4% paraformaldehyde solution (200 μL, ThermoScientific); the plates were then incubated at 37 °C for 30 min. Finally, all the fixed spheroids were stored at 4 °C for further analysis with SWIR imaging. Furthermore, brain tissue slices of 1 mm thickness were prepared and overlaid with capillaries filled with PbS QDs (4 mg mL^−1^) and AFt-PbS (2.5 mg mL^−1^). Following NIR imaging, a concentration-dependent intensity profile was generated and analyzed.

### 2.6 High resolution transmission electron microscopy (HR-TEM)

PbS QD (0.5 mg mL^−1^) and AFt-PbS-TMZ (AFt concentration 1 μM) samples were diluted in Millipore water (PbS QDs) and 0.1 M sodium acetate buffer (pH 5.5) (AFt samples), respectively. The solutions were drop-cast on a graphene oxide copper grid and dried under vacuum for 10 min. Negative staining with uranyl acetate (2%) was used for protein formulations. Images were acquired using a JEOL 2100Plus electron microscope operating at 200 kV.

### 2.7 Non-denaturing polyacrylamide gel electrophoresis (native-PAGE)

Native-PAGE was used to analyze AFt samples. Samples of each solution (18 μL) were loaded onto native-PAGE gels (4–16% bis-tris pre-cast gels) and run at 150 V for 1 hour (h) and at then 250 V for 1 h. Subsequently, the gel was stained with BlueSafe protein stain (Thermo Scientific) and washed with deionized water.

### 2.8 Drug loading (DL), encapsulation efficiency (EE), and protein yield (PY)

The concentration of AFt was measured using Bradford assay (Bradford reagent (Sigma)^33^) whereas TMZ inside AFt cages was measured using ultraviolet visible (UV-Vis) spectroscopy (Agilent Cary UV-Vis Multicell Peltier). AFt-TMZ encapsulation efficiency was assessed using a direct technique *via* UV-spectroscopy measurement at *λ* = 330 nm (TMZ) and *λ* = 265 nm (5-amino-imidiazole-4-carboxoamide (AIC)) whereas AFt-PbS-TMZ encapsulation efficiency was determined using an indirect method *via* UV-spectroscopy measurement. The following equations were used:1
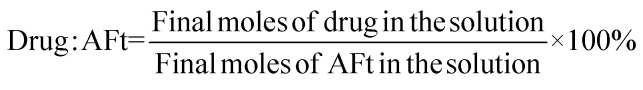
2
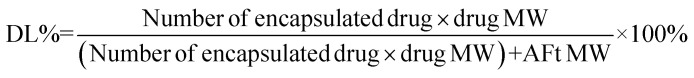
3
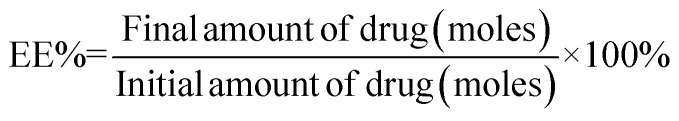
4
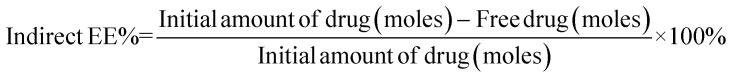
5
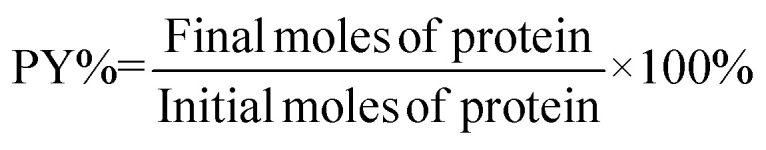


### 2.9 Cell culture studies

Cell culture studies were conducted using GBM cell lines, including U373M (MGMT-transfected), U373V (vector control), U87MG (MGMT low) and non-tumourigenic cell lines, including the foetal lung fibroblast line MRC-5 and human astrocytes (frontal lobe). U87MG and MRC-5 cells were purchased from the American Type Culture Collection (ATCC), while isogenic U373M and U373V were gifted by Schering Plough Corporation and human astrocytes were purchased from ScienCell Research Laboratories. U373M and U373V cells were cultured in RPMI 1640 medium supplemented with 10% foetal bovine serum (FBS), 1% v/v non-essential amino acids (NEAA), 50 μg mL^−1^ gentamicin, and 400 μg mL^−1^ G418 (Corning). U87MG cells were cultured in MEM supplemented with 10% FBS and 1% l-glutamine, while MRC-5 cells were cultured in MEM with 10% FBS, 1% v/v NEAA, 1% v/v penicillin/streptomycin, 2 mM l-glutamine, and 10 mM HEPES buffer. Human astrocytes were cultured in poly-l-lysine-coated (ScienceCell™, 10 mg mL^−1^, CAT: 0413) T75 flasks and incubated at 37 °C overnight. The astrocytes were maintained in complete astrocyte medium (ScienceCell™, CAT: 1801), and passaged using trypsin/EDTA (T/E) solution 0.05% (ScienceCell™, CAT: 0183) and T/E neutralization solution (TNS, ScienceCell™, CAT:0113) according to manufacturers' recommendations. Human astrocytes were used up to passage number 10. All the cell lines were incubated in 5% CO_2_ at 37 °C.

### 2.10 3-(4,5-Dimethyl thiazolyl-2)-2,5-diphenyltetrazolium bromide (MTT) assay

*In vitro* growth inhibitory studies were performed using 3-(4,5-dimethylthiazolyl-2)-2,5-diphenyltetrazolium bromide (MTT) assays, which measure viable cell metabolic activity by quantifying the optical density of the formazan product at 570 nm. Briefly, GBM cells were seeded in 96-well plates at a density of 650 cells/well in 180 μL medium, while MRC-5 cells were seeded at a density of 400 cells/well. After 24 h to allow for cell attachment, cells were treated with 20 μL test compounds (AFt, TMZ, PbS QDs, TMZ + PbS QDs, AFt-PbS, AFt-TMZ, and AFt-PbS-TMZ) at 10× final concentrations and incubated for 6 days. Following incubation, the MTT reagent (50 μL, 400 mg mL^−1^) was added into each well, and the plates were incubated at 37 °C in a humidified atmosphere with 5% CO_2_ for 2 h to allow for formazan production. Then, the aqueous medium was aspirated, and the formazan product was solubilized by adding 150 μL/well of DMSO. The plates were placed on a shaker for 5 min before the absorbance of each well was read at 570 nm using a PerkinElmer Envision plate reader. The 50% growth inhibition (GI_50_) and the corresponding half-maximal inhibitory concentration (IC_50_) values were determined for each formulation.

### 2.11 Two-dimensional (2D) presto blue cell viability assay

U373M, U373V, U87MG (650 cells/well) and MRC-5 (400 cells/well) and human astrocytes (5000 cells/well) were seeded in 96 well plates in 81 μL of cell culture medium and incubated overnight at 37 °C in a humidified atmosphere with 5% CO_2_. Cells were treated at 10× final concentrations of test compounds (AFt, TMZ, PbS QDs, TMZ + PbS QDs, AFt-PbS, AFt-TMZ, and AFt-PbS-TMZ) and incubated for 6 days. PB cell viability reagent (10 μL; 10×) was then added to each well (final volume 100 μL) according to the manufacturer's protocol (CAT:A13262). The 96-well plates were then wrapped in foil to protect the cells from light and incubated at 37 °C in a humidified atmosphere with 5% CO_2_ for 3 h. The absorbance of the reagent was measured at 570 nm, using 600 nm as a reference wavelength, using a PerkinElmer Envision plate reader. IC_50_ values were calculated for each test agent. 2D cell viability was assessed by both MTT and PB methods.6
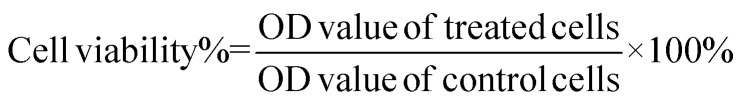


### 2.12 Western blotting

The expression levels of TfR1, MGMT, and glyceraldehyde 3-phosphate dehydrogenase (GAPDH) in 2D cell cultures were evaluated using western blotting. Protein lysates were collected from U373M, U373V, U87MG, human astrocytes, and MRC-5 cells, and their concentrations were quantified using the Bradford assay. SDS-PAGE was used to resolve 50 μg of protein from each lysate, which was then transferred to nitrocellulose membranes (GE Healthcare Life Sciences). The membranes were blocked with 5% non-fat milk solution at room temperature (RT) for 1 h. Following blocking, the membranes were incubated overnight at 4 °C with primary antibodies: recombinant anti-human TfR1 monoclonal antibodies 1 : 1000; ThermoFisher (CAT:136800), anti-human MGMT monoclonal antibodies (1 : 500; Invitrogen (MA3-16537), and anti-human GAPDH monoclonal antibodies (1 : 2000; Sigma Aldrich (CAT:G8795). Subsequently, the membranes were incubated at RT for 1 h with a secondary antibody, goat anti-mouse IgG (H + L) superclonal secondary antibodies (1 : 2500/1 : 2000; ThermoFisher (CAT:A28177). Protein bands were visualized using a C-DiGit blot scanner (LI-COR Biosciences) after 5 min of incubation with ECL (GE Healthcare) substrate.

### 2.13 Three-dimensional (3D) tumour spheroids

U87MG tumour spheroids were grown in ultra-low attachment (ULA) plates (ThermoFisher). Cells were seeded in 90 μL of cell culture medium at an optimized density (3000 cells/well) including 6 replicates per experiment. The plates were centrifuged (300*g*, 5 min) and then incubated overnight at 37 °C in a humidified atmosphere with 5% CO_2_. The following day, U87MG tumour spheroids were treated with 10 μL of 10× final concentration test agents (AFt, 0.01–1 μM; TMZ, 0.001–1000 μM; AFt-TMZ, 0.01–200 μM; PbS QDs, 0.01–100 μg mL; AFt-PbS, 0.01–100 μg mL; AFt-PbS-TMZ, 1–200 μM) for 6 days. The tumour spheroids were photographed; ImageJ software was used to measure the mean spheroid diameter at day 1 and day 7 employing horizontal (d1) and vertical (d2) diameters. The spheroid volume was calculated for each spheroid using the following equation:7
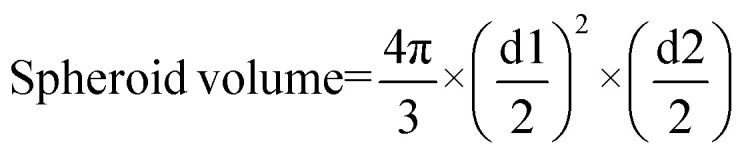


PB assays were used to determine spheroid cell viability by adding 11 μL of PB reagent to each well; plates were wrapped in foil and incubated for 6 h at 37 °C. The absorbance was then measured using a PerkinElmer Envision plate reader, and IC_50_ values were determined. The mean spheroid volume and the percentage cell viability were compared at the end of the studies to evaluate the effects of the test agents.

### 2.14 Statistical analysis

All experiments were performed on at least three independent occasions (*N* = 6 internal replicates) and data are expressed as mean ± standard deviation (SD). Group differences were analyzed using GraphPad Prism. Statistical analysis included one-way analysis of variance (ANOVA) followed by the Holm–Sidak method for comparisons involving three or more groups (*n* ≥ 3) and *t*-tests for comparisons between two groups (*n* = 2). Statistical significance was reported as follows: not significant (ns) *p* > 0.05, significant (*) *p* < 0.05, (**) *p* < 0.01, (***) *p* < 0.001, and (****) *p* < 0.0001.

## 3 Results and discussion

### 3.1 Co-encapsulation of QDs and TMZ

**Fig. 1:**
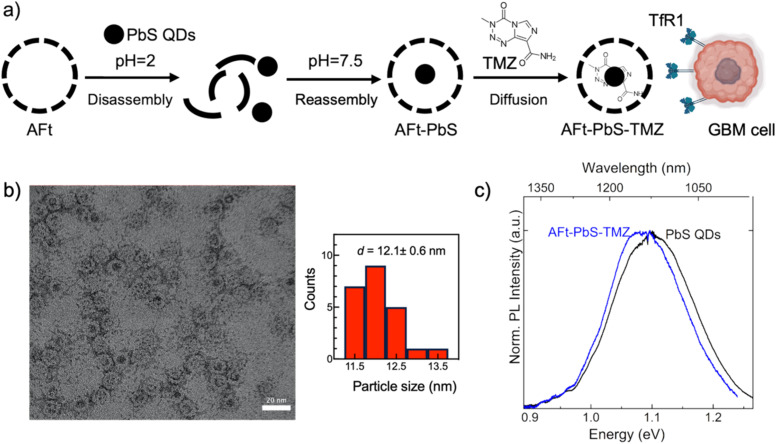
(a) Schematic illustration of the encapsulation process in the development of the theranostic agent AFt-PbS-TMZ for targeting of TfR1, overexpressed in cancer cells. (b) Representative HR-TEM image of AFt-PbS-TMZ, negatively stained with uranyl acetate and a size distribution histogram is shown in the inset. (c) Room temperature photoluminescence (PL) spectra of PbS QDs and AFt-PbS-TMZ.

To integrate imaging and therapeutic agents within a single structure, PbS QDs were encapsulated into horse spleen AFt cages using the disassembly–reassembly method to form AFt-PbS. This was followed by encapsulation of TMZ through passive diffusion to form AFt-PbS-TMZ (Fig. 1a). PbS QDs used for encapsulation were synthesized in aqueous solution with an average diameter, *d* = 4.0 ± 0.5 nm and PL emission centred at 1118 nm at room temperature. (SI Fig. S2, and 1c). Individual agents, PbS QDs and TMZ, were also encapsulated into AFt cages as controls, using reassembly and diffusion routes, respectively. All encapsulated agents were comprehensively characterised to probe their morphology and composition. Dynamic light scattering (DLS) and native-PAGE studies confirmed that in all formulations, AFt-PbS, AFt-TMZ, and AFt-PbS-TMZ, the AFt cage retained its size (12.3 ± 0.7 nm, 12.0 ± 0.8 nm, and 14.3 ± 0.5 nm, respectively) and the surface charge (−4.1 ± 0.3, −4.5 ± 0.3, and −4.2 ± 0.4 mV, respectively), confirming that the surface of the AFt is not altered by the encapsulation process and that no agents are attached to the protein surface (SI Fig. S3a and S3b). Both, the nanoscale size and surface charge of AFt are important for the development of theranostic agents, as this will define the cellular uptake of the agents and *in vivo* circulation times. High-resolution transmission electron microscopy (HR-TEM) images of negatively stained AFt-PbS-TMZ (Fig. 1b) were consistent with DLS measurements, revealing intact AFt shells with an external diameter of 12.1 ± 0.6 nm. Following encapsulation, PbS QDs retained their optical properties, with only a small redshift in the PL peak position to *λ*_em_ = 1131 nm (Fig. 1c), likely due to a reduction in the strength of quantum confinement.^34^

The encapsulation of TMZ in AFt-PbS was investigated using UV-Vis spectroscopy by measuring the intensity of the TMZ absorption peak at *λ* = 330 nm. The results showed that for the AFt : TMZ ratio of 1 : 400 molecules used in encapsulation process, the EE% of TMZ was 74.4 ± 11.2% corresponding to 309 ± 49 molecules per AFt cage in the AFt-PbS-TMZ formulation (Table 1). In our work, only one PbS QD can be encapsulated within the AFt interior (8 nm diameter); hence there is sufficient cavity volume for entrapment of small molecules. We envisaged that the small molecular size of TMZ (194.1 g mol^−1^, ∼309 molecules per cage) enables encapsulation of a significantly higher number of molecules compared to larger drugs, such as paclitaxel (853.9 g mol^−1^, ∼60 molecules per cage), DOX (543.5 g mol^−1^, ∼28 molecules per cage), or Phortress (386.5 g mol^−1^, ∼130 molecules per cage).^35–37^ As the duration of the encapsulation process (∼2.5 h at ∼pH 7.5) was longer than the TMZ half-life (1.8 h at pH 7.4), the presence of its degradation product, metabolite AIC, was also monitored at *λ* = 265 nm. However, minimal degradation was observed, indicating that an optimal encapsulation protocol was established.

**Table 1:** Summary of encapsulation efficiency (EE%), drug loading (DL%), and protein yield (PY%) of AFt nanoparticles (mean ± SD, *n* = 6)

Nanoparticle	AFt/TMZ ratio	EE (%)	DL (%)	PY (%)	Number of molecules per AFt
AFt-TMZ	1 : 800	73.1 ± 12.1	16.8 ± 0.9	81.6 ± 12.9	516 ± 82
AFt-PbS-TMZ	1 : 400	74.4 ± 11.2	13.1 ± 3.2	80.1 ± 16.8	309 ± 49

Comparable TMZ release was observed from AFt-PbS-TMZ (∼70%) and from AFt-TMZ (∼78%)^7^ after 24 h at pH 5.5 (SI Fig. S4a). In contrast, at pH 7.4, retention of TMZ was significantly higher in AFt-PbS-TMZ, with only 33% release after 24 h compared to 82% from AFt-TMZ (SI, Fig. S4b). We envisage that at higher pH values, there is a stronger interaction between the QD surface and TMZ, as a higher partial charge on the functional groups of TMZ may interact with the surface charge on the QD surface. While it is not possible to directly probe the interactions, it is likely that the NH_2_-group of TMZ can interact with free Pb(ii) coordination sites on the QD surface or form H-bonds with the capping thioglycerol corona. Notably, that NH_2_ groups are commonly used for ligand binding to QDs. Considering the lower stability of TMZ at higher pH, binding to MTIC is possible and may confer additional stability, as suggested previously for binding with CuNPs.^38^ TMZ may bind to the corona of QDs or π-stack with aromatic residues (tryptophan, tyrosine, and phenylalanine) on the interior surface and hydrophobic pores of AFt. The interior surface of the AFt cage is rich in hydrogen bonding groups including many carboxylates (Asp/Glu) that have the potential to interact with the two hydrogen bond donors (–NH_2_) and five strong hydrogen bond acceptors (N and O) on each TMZ molecule. Previously, TMZ binding to human serum albumin (HSA) was shown, extending TMZ half-life *in vivo* with delayed hydrolysis.^39^ Similarly, enhanced TMZ stability was found when TMZ bound to carrier systems.^40,41^ Altogether, this indicates an additional benefit of co-encapsulation of QDs^20,42^ with TMZ, which could offer a route to pH induced control of the rate of TMZ release and its mechanism merits detailed studies.

### 3.2 Two-dimensional (2D) *in vitro* cellular studies

The growth inhibitory effects of AFt-PbS-TMZ were examined using both MTT and PB assays in U373M, U373V, and U87MG GBM cells as well as in MRC-5 fibroblasts and human astrocytes which served as representatives of normal cells. Comparison of the results of these two assays enabled variations in cellular metabolism, enzyme expression and redox conditions across the different cell lines to be accounted for. U373M, U373V, and U87MG cells were specifically selected for study due to their varying MGMT levels which are associated with TMZ resistance.^7^

**Fig. 2:**
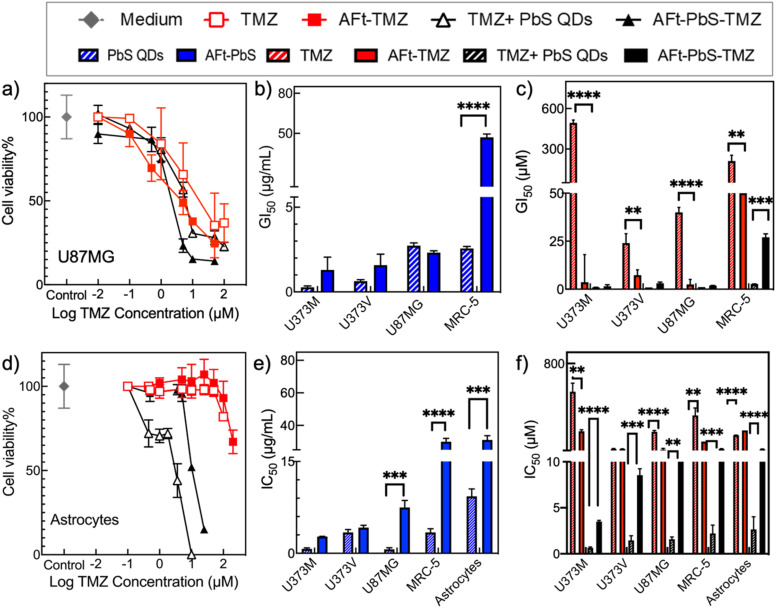
*In vitro* growth inhibitory studies of GBM cell lines and non-tumourigenic cells in 2D monolayers. (a) Representative MTT graph of U87MG cells after 6-day treatment with TMZ, AFt-TMZ, TMZ + PbS QDs and AFt-PbS-TMZ. (b) The comparative GI_50_ (PbS QDs) values (μg mL^−1^) for PbS QDs and AFt-PbS obtained from MTT on non-tumourigenic fibroblasts (MRC-5) and glioblastoma cell lines (U373M, U373V, and U87MG). (c) The summary of GI_50_ (TMZ) values (μM) for TMZ, AFt-TMZ, TMZ + PbS QDs, and AFt-PbS-TMZ obtained from MTT on GBM and MRC-5 cells. (d) Representative PB graph of human astrocytes after 6-day treatment with TMZ, AFt-TMZ, TMZ + PbS QDs and AFt-PbS-TMZ. (e) Comparison of IC_50_ (PbS QDs) values (μg mL^−1^) for PbS QDs and AFt-PbS in all studied cell lines with PB assay. (f) Comparison of IC_50_ (TMZ) values (μM) for TMZ, AFt-TMZ, TMZ + PbS QDs and AFt-PbS-TMZ in all studied cell lines with PB assay. Data are presented as mean ± SD of samples from three independent experiments. (*n* = 3 and *N* = 6).

Cells were exposed to serial dilutions of the nanoformulations for a period of six days to allow TMZ to exert its cellular effects through DNA methylation, MMR activation, and two cell cycles. The lowest GI_50_ value was observed following treatment of U87MG cells with the co-encapsulated formulation of PbS QDs and TMZ in AFt (Fig. 2a), compared to PbS QDs, naked TMZ or AFt formulations (Fig. 2b and c). Encapsulation of PbS QDs into AFt cages (AFt-PbS) reduced (∼20-fold) the cytotoxicity of PbS QDs in non-tumourigenic fibroblasts while retaining their activity against GBM cells (Fig. 2b). This observation aligns with previous reports in colorectal and breast cancer cells, where the cytotoxic effect was attributed to ROS-induced apoptosis.^26,43^ For both individual and co-encapsulated agents, the presence of the AFt shell significantly enhanced the potency of TMZ and PbS QDs against cancer cells, with significantly reduced GI_50_ values (*p* < 0.01) observed in all GBM cell lines, compared to non-encapsulated agents. The MTT results showed that U373M cells (GI_50_ (TMZ) = 490 ± 20 μM) were markedly less sensitive to TMZ compared to U373V (GI_50_ (TMZ) = 24 ± 5 μM) and U87MG (GI_50_ (TMZ) = 40 ± 3 μM) cells, corroborating a previous report.^7^ In contrast, AFt-encapsulated TMZ yielded GI_50_ values <10 μM in TMZ-resistant U373M cells, demonstrating that AFt encapsulation of TMZ effectively overcomes clinical resistance mediated by *O*6MeG repair *via* MGMT. Further enhancement of therapeutic activity was observed with the co-encapsulated AFt-PbS-TMZ formulation, with GI_50_ (TMZ) = 1.5 ± 0.8 μM in U373M, GI_50_ (TMZ) = 3.1 ± 0.6 μM in U373V, and GI_50_ (TMZ) = 1.9 ± 0.1 μM in U87MG GBM cell lines (Table 2).

**Table 2:** Summary of GI_50_ and IC_50_ for all formulations in the studied cell lines (mean ± SD, *n* = 3)

	Formulation					
	PbS QDs (μg mL^−1^)	AFt-PbS (μg mL^−1^)	TMZ (μM)	AFt-TMZ (μM)	TMZ + PbS (μM)	AFt-PbS-TMZ (μM)
**Cell lines**	**GI**_**50**_ **(PbS QDs)**		**GI**_**50**_ **(TMZ)**			
U373M	0.3 ± 0.1	1.3 ± 0.8	490 ± 20	3.7 ± 1.4	0.9 ± 0.1	1.5 ± 0.8
U373V	0.6 ± 0.1	1.6 ± 0.7	24 ± 5	7.3 ± 2.7	0.9 ± 0.1	3.1 ± 0.6
U87MG	2.7 ± 0.2	2.3 ± 0.1	40 ± 3	2.5 ± 2.6	1.1 ± 0.1	1.9 ± 0.1
MRC-5	2.6 ± 0.1	47 ± 3	210 ± 40	50 ± 1	2.7 ± 0.1	27 ± 2

	**IC**_**50**_ **(PbS QDs)**		**IC**_**50**_ **(TMZ)**			
U373M	0.7 ± 0.2	2.7 ± 0.3	550 ± 80	190 ± 10	0.6 ± 0.1	3.5 ± 0.1
U373V	3.4 ± 0.5	4.2 ± 0.4	33 ± 5	33 ± 2	1.5 ± 0.5	8.5 ± 0.7
U87MG	0.7 ± 0.3	7.5 ± 1.2	190 ± 10	28 ± 11	1.6 ± 0.3	11 ± 3
MRC-5	3.4 ± 0.6	30 ± 2	330 ± 70	>100	2.2 ± 0.9	33 ± 4
Astrocyte	9.3 ± 1.2	31 ± 3	160 ± 2	>200	2.6 ± 1.4	31 ± 4

Encapsulated agents exhibited reduced cytotoxicity in non-tumourigenic fibroblasts (MRC-5) and human astrocytes compared to cancer cells. For example, GI_50_ (TMZ) > 2.5 μM was observed for TMZ + PbS QDs and GI_50_ (TMZ) > 25 μM for AFt-PbS-TMZ in MRC-5 cell lines, demonstrating that AFt encapsulation provides protection against toxicity in non-cancer cells. The results from MTT studies were corroborated by the PB assay, which showed higher IC_50_ values for all encapsulated agents in human astrocytes (Fig. 2d–f, and Table 2). The AFt vehicle alone was non-toxic across all studied cell lines, and for all nanoformulations, the same trends were observed in both MTT and PB assays (SI Fig. S5).

**Fig. 3:**
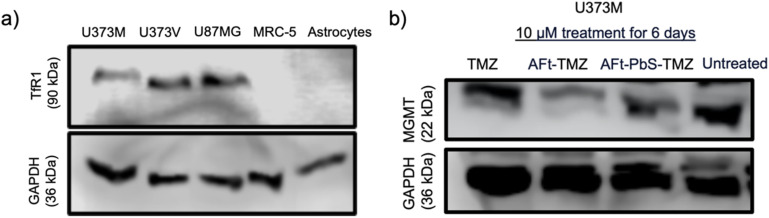
(a) Western blot analysis of TfR1 and GAPDH (loading control) expressions in all studied cell lines. (b) Down-regulation of MGMT expression in U373M cells exposed to 10 μM TMZ, AFt-TMZ and AFt-PbS-TMZ for 6 days.

The selective anticancer effect of all AFt nanoformulations in GBM cells compared to non-tumourigenic cells (astrocytes and MRC-5) is likely facilitated by specific targeting to TfR1 receptors overexpressed in carcinoma cells.^21^ Our western blot results (Fig. 3a) confirm high TfR1 expression in the studied cancer cells compared to MRC-5 fibroblasts and human astrocytes. Moreover, we provide evidence to support delivery of AFt-encapsulated cargo *via* TfR-1-mediated cellular internalisation. Fig. 3b demonstrates significant (*p* < 0.001 for AFt-TMZ and *p* < 0.05 for AFt-PbS-TMZ) downregulation of the direct repair protein MGMT in U373M cells following exposure to AFt-TMZ or AFt-PbS-TMZ respectively. This finding indicates that the TMZ burden delivered to U373M cells overwhelms the suicide repair protein that removes *O*6-methylguanine lesions and confers TMZ-resistance. In line with reduced MGMT expression in U373M cells (Fig. 3b), we observed their enhanced sensitivity to TMZ when delivered encapsulated within AFt (Fig. 2c, f and Table 2).

### 3.3 Evaluation of theranostic capabilities of AFt-PbS-TMZ in 3D spheroids

**Fig. 4:**
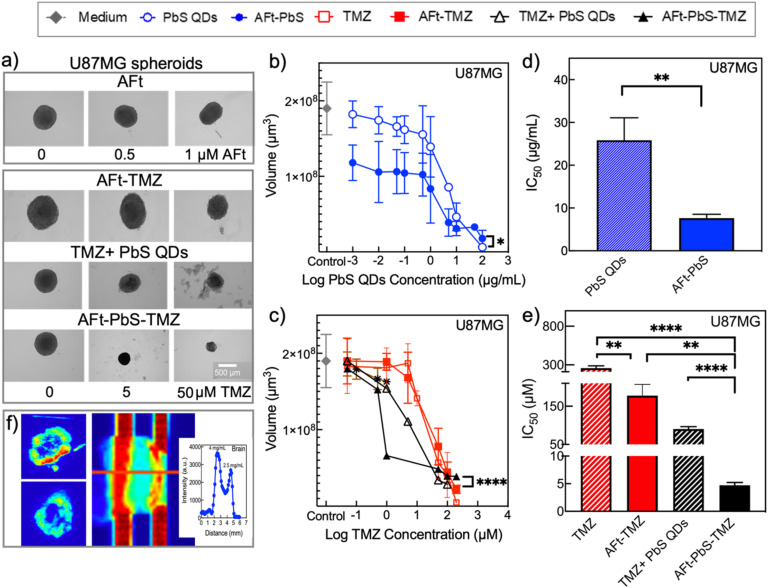
The effect of naked and loaded formulations on 3D U87MG spheroids. (a) Representative optical images of 3D U87MG spheroids on day 7 including AFt alone (vehicle) for the control (0), 0.5 μM and 1 μM treatments and AFt-TMZ, TMZ + PbS QDs, and AFt-PbS-TMZ for the control (0), 5 μM and 50 μM treatments (the scale bar is 500 μm). (b) Spheroid volume on day 7 treated with PbS QDs (0.001–100 μg mL^−1^) and AFt-PbS (0.001–100 μg mL^−1^). (c) Spheroid volume on day 7 treated with TMZ, AFt-TMZ, TMZ + PbS QDs and AFt-PbS-TMZ. The comparison IC_50_ values of PbS QDs and AFt-PbS (d) and TMZ, AFt-TMZ, TMZ + PbS QDs, and AFt-PbS-TMZ (e) on 3D U87MG spheroids. Data are presented as mean ± SD of samples from three independent experiments; (*n* = 4, *N* = 6). (f) Near-infrared imaging of 50 μg mL^−1^ PbS QD- and AFt-PbS-TMZ-treated U87MG spheroids (785 nm laser at 1.5 W, emission filter 980 nm LP, exposure time: 0.03 s, histogram stretching: 20–5000), (inset) PbS QD (4 mg mL^−1^) and AFt-PbS (2.5 mg mL^−1^) loaded capillary tubes overlaid with brain tissue slices and their corresponding concentration-dependent intensity profiles (785 nm laser at 20% power, 1000 nm LP, exposure time:0.1 s, and histogram stretching: 1000–5000).

To evaluate the potential of our nanocomposites for theranostic applications, we used 3D spheroids, which better represent tumours *in situ* by exhibiting intercellular communication, nutrient and oxygen gradients and cell polarity that are lacking in 2D cultures. U87MG tumour spheroids were grown in ULA plates, with an average diameter of 400 ± 101 μm, and exposed to all nanoformulations for a period of six days (Fig. 4a and SI S6a). AFt alone (vehicle) was non-toxic at the concentrations utilized in AFt-PbS-TMZ (see SI Fig. S6b, 4a and Table 3) in 3D spheroids. Consistent with the results in 2D cultures, the treatment with AFt-encapsulated agents over 6 days led to a concentration-dependent decrease of the spheroid diameter, volume and cell viability (Fig. 4b and c). Treatment with AFt-PbS led to a significant decrease (*p* < 0.05) in spheroid volume at lower concentrations (<10 μg mL^−1^) compared to higher concentrations (>25 μg mL^−1^) required for a similar effect with PbS QDs alone. The volumes of spheroids exposed to TMZ + PbS QDs and AFt-PbS-TMZ decreased by 50% following exposure to treatment concentrations between 5 and 10 μM and between 1 and 5 μM, respectively (*p* < 0.0001). Among all the treatments, the co-encapsulated AFt-PbS-TMZ formulation demonstrated the most potent inhibitory effect on the spheroid growth rate.

**Table 3:** The summary of AFt, TMZ and PbS QD concentrations in the AFt-PbS-TMZ formulation

	AFt	TMZ	PbS QDs
AFt-PbS-TMZ	0.28 μM	2338 ± 451 μM	2100 μg mL^−1^

Treatment with AFt-nanoformulations resulted in lower IC_50_ values compared to unencapsulated agents. For example, naked PbS QDs exhibited an IC_50_ of 26 ± 5 μg mL^−1^, over 3-fold higher than that of AFt-PbS (8 ± 1 μg mL^−1^) (Fig. 4d). Similarly, the IC_50_ of TMZ was reduced from 260 ± 30 μM to 180 ± 30 μM following AFt encapsulation. Moreover, co-encapsulating TMZ and PbS QDs in AFt nanocages (AFt-PbS-TMZ) reduced the IC_50_ to 5 ± 1 μM (about 20-fold decrease (*p* < 0.0001) compared to naked TMZ mixed with PbS QDs (90 ± 6 μM) (Fig. 4e). The results in 3D U87MG spheroids suggest that co-encapsulated AFt-PbS-TMZ is the most effective formulation for inducing significant reduction in spheroid volumes and increasing anti-tumour activity. Therefore, AFt encapsulation offers a promising strategy to decrease the required concentration of TMZ, enhance targeted drug delivery to tumour cells, and improve drug availability in the tumour microenvironment.

In 3D spheroids treated with 50 μg mL^−1^ (equivalent to ∼50 μM based on TMZ) AFt-PbS-TMZ, PL was detected on the surface (Fig. 4f, and SI S7) and within the interior of the spheroids, demonstrating their ability to penetrate deep within the spheroid structure. Importantly, detectable PL was recorded at room temperature for QD solutions at a concentration of 0.083 mg mL^−1^ which is below the GI_50_ value (SI Fig. S8), clearly indicating realistic potential for using QDs as NIR imaging probes. Moreover, capillary tubes filled with PbS QD (4 mg mL^−1^) and AFt-PbS (2.5 mg mL^−1^) solutions were tested at 1 mm depth (Fig. 4f). The NIR emission of PbS QDs enabled imaging below the surface, with concentration-dependent PL signals measurable under brain slices (Fig. 4f inset). At a depth of 1 mm, PbS QDs (4 mg mL^−1^) exhibited a PL intensity of 3500, while AFt-PbS (2.5 mg mL^−1^) demonstrated a lower intensity of 2700 (histogram range: 1000–5000). These findings further suggest that formulations containing PbS QDs can facilitate NIR imaging through brain tissue, with imaging intensity and clarity being influenced by depth and concentration.

### 3.4 Discussion

Although TMZ is used in the treatment of GBM and has been shown to extend median survival from ∼4 months to ∼16 months, it remains far from offering a cure for GBM patients, mostly due to systemic toxicities and TMZ resistance.^7^ Theranostic strategies are being developed to create multifunctional nanoformulations that combine imaging capabilities of NIR-emitting PbS QDs and therapeutic activity of TMZ, along with the innate cancer (TfR1)-targeting ability of the AFt carrier.^7,22^ AFt encapsulation increased therapeutic effects of drugs/imaging agents in GBM,^7^ colorectal,^26^ and breast^43^ cancer cells, as demonstrated in 2D monolayer cell cultures. Our results corroborate selective and enhanced anticancer activity in 2D and 3D GBM models, resulting from natural targeting ability of AFt towards TfR1 receptors overexpressed on cancer (GBM) cells.^7,21,22^ Of particular note, AFt-mediated delivery of TMZ was able to overcome TMZ-resistance mediated by MGMT repair in U373M cells, which we hypothesise, occurs *via* enhanced TMZ delivery; this is supported by the downregulation of this suicide repair protein in cells exposed to AFt-TMZ formulations. The small size, negative surface charge and bioavailability of AFt are beneficial for drug delivery to GBM cancers due to TfR1-mediated endocytosis, and are expected to reduce immune responses and non-specific cellular uptake.^23^

However, high failure rates in clinical trials for GBM treatment are frequently attributed to BBB impermeability.^44^ Numerous strategies have been attempted to permeabilize or facilitate transcytosis across the BBB, including the exploitation of BBB endothelial expression of TfR1.^44^ For instance, drugs such as paclitaxel have been conjugated to transferrin to enhance the drug delivery to the brain by receptor mediated endocytosis.^45^ Additionally, the failure rates may be partly attributed to the reliance on 2D models in drug discovery studies. Thus, 3D tumour spheroids have an increasingly importance role in *in vitro* evaluation. In this work, the significant reduction in spheroid volume and cell viability observed in 3D cultures is more representative of the *in vivo* environment rather than 2D models since spheroids contain necrotic, proliferating and non-proliferating cells which more accurately match the structure and heterogeneity of solid tumours.^46^ Our results demonstrate that 3D spheroids are less sensitive to treatments yielding higher IC_50_ values compared to 2D models. Altogether, these data demonstrate that co-encapsulation of PbS QDs and TMZ into AFt cages elicits greater growth inhibitory effects in 3D U87MG spheroids than other AFt formulations and allows imaging in the NIR-II region. Contributing to enhanced resistance may be the compact structure of 3D spheroids including barriers and increased intracellular and extracellular cell signalling in the tumour microenvironment.^30^ AFt-PbS-TMZ demonstrated lower IC_50_ values in 3D cultures compared to 2D cultures, similar to the results with folic acid-functionalized silver and upconverting nanoparticles, where greater (20%) uptake in 3D cultures was observed.^47^ This may be attributed to differences in several factors including the pH levels, oxygen availability, ECM, metabolic activity, and cell signalling of spheroid structures.^30^

Furthermore, QDs offer an exciting prospect for cancer imaging thanks to their high fluorescence emission, photostability, and narrow emission spectrum.^18^ In this work, we have demonstrated that PbS QD emission at SWIR wavelengths allows imaging from within 3D spheroids. Overall, these data suggest that AFt-PbS-TMZ formulations might be beneficial in GBM (and more broadly cancer) theranostic applications as a surveillance tool for monitoring and managing cancer progression. In addition, the study provides a proof of concept (PoC) for the future development of theranostic protein nanoparticles, and their potential application for brain tumours, given their high PL activity and resolution. Current trends highlight the importance of 3D *in vitro* studies, as these inform and improve *in vivo* outcomes and can reduce the number of animals used. Our studies reported here will underpin detailed *in vivo* evaluation of the activity of these theranostic nanoplatforms, in terms of BBB penetration, targeting of brain cancer cells, imaging acuity and therapeutic efficacy.

## 4. Conclusions

Conventional treatment and diagnostic methods for GBM have major limitations. Therefore, improved approaches for the diagnosis and treatment of GBM are required. PbS QDs, in particular, have shown promise for bio-imaging but need appropriate nanocarriers to overcome cytotoxicity issues. The unique properties of AFt make it attractive for therapeutic, imaging and diagnostic (theranostic) applications to address these problems. We investigated whether co-encapsulation of TMZ and PbS QDs into AFt cages has the potential to overcome TMZ resistance, reduce toxicity in healthy cells, and enable imaging of GBM. In this study, the activity of PbS QDs against non-cancer cells was reduced by horse spleen AFt encapsulation. Additionally, TMZ encapsulation in AFt potentiates its activity in malignant GBM 2D and 3D cultures. Co-encapsulation of PbS QDs and TMZ into AFt cages (AFt-PbS-TMZ) offers the potential to direct theranostic molecules to the tumour site, enable tumour imaging, enhance the activity of the therapeutic moiety and protect normal tissues from toxic effects of imaging and therapeutic agents. AFt is an exciting nano-sized vehicle worthy of further development for cancer theranostic purposes to target, image and deliver drugs to cancer cells, due to its ability to target cancer cells and reduce toxicity towards non-cancerous cells.

## Conflicts of interest

There are no conflicts to declare.

## Supplementary Material

NA-007-D5NA00557D-s001

## Data Availability

All raw data contributing to the results reported in the manuscript and its supporting information are stored in the University of Nottingham's OneDrive cloud storage. They are freely available upon request from the corresponding author. Supplementary information: details of sample characterization, experimental results of *in vitro* cell culture studies on all cell lines and text agents, and additional results of optical studies and SWIR imaging. See DOI: https://doi.org/10.1039/d5na00557d.
