# Supplementary material for: Co-encapsulation of temozolomide and PbS quantum dots in apoferritin for transferrin receptor 1 targeting, imaging and treatment of glioblastoma
Source: Nanoscale Adv. 2025 Oct 3;7(21):6808–18. doi: 10.1039/d5na00557d (PMC12516861; doi:10.1039/d5na00557d)
Supplement: NA-007-D5NA00557D-s001 [file NA-007-D5NA00557D-s001.pdf]

Supplementary Information (SI) for Nanoscale Advances.
This journal is © The Royal Society of Chemistry 2025

SUPPLEMENTARY INFORMATION

Co-encapsulation of Temozolomide and PbS Quantum Dots in Apoferritin for Transferrin Receptor 1 Targeting, Imaging and Treatment of Glioblastoma

Reyhan Dilsu Colpan,[a] Ellie B. Ward,[b] Dongling Zhang,[d] Erica Ling,[d] Umar Iqbal,[d] Maria Moreno,[d] Neil R. Thomas,[c] Lyudmila Turyanska,[b] Tracey D. Bradshaw[a]*

[a] *Biodiscovery Institute, School of Pharmacy, University of Nottingham, NG7 2RD, UK,*
[b] *Faculty of Engineering, University of Nottingham, Nottingham NG7 2RD, UK,*
[c] *Biodiscovery Institute, School of Chemistry, University of Nottingham, NG7 2RD, UK,*
[d] *National Research Council Canada, Ottawa, Canada.*

Corresponding author(s): Tracey.Bradshaw@nottingham.ac.uk

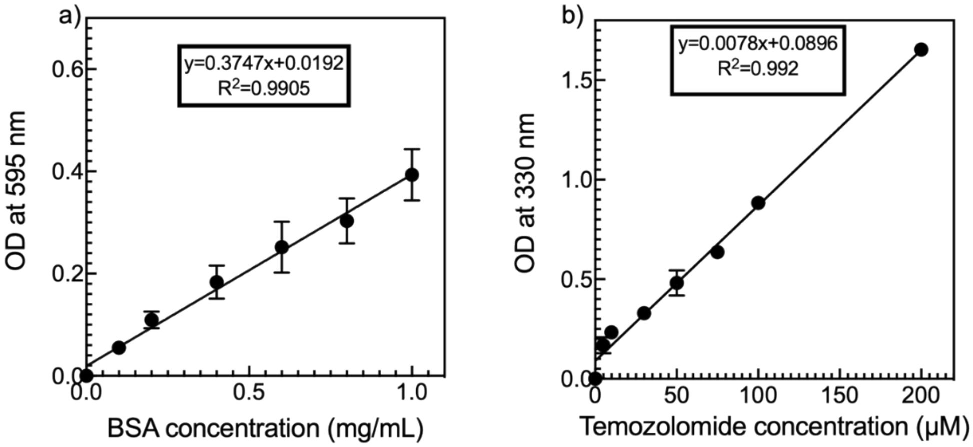

Figure S1. Standard curves of bovine serum albumin (BSA) **(a)** and TMZ **(b).**

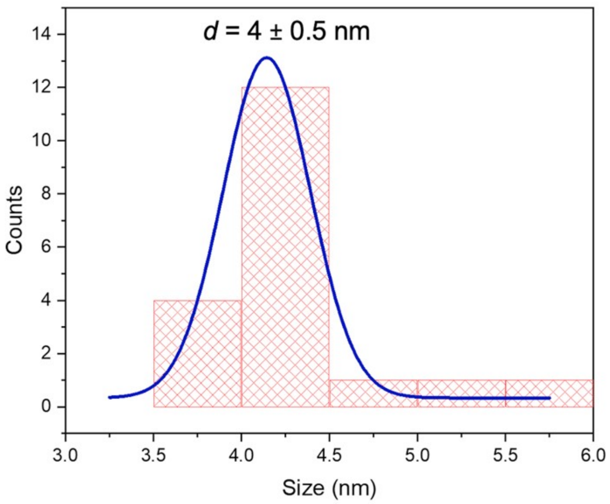

Figure S2. PbS QDs average size distribution graph obtained from HR-TEM analysis.

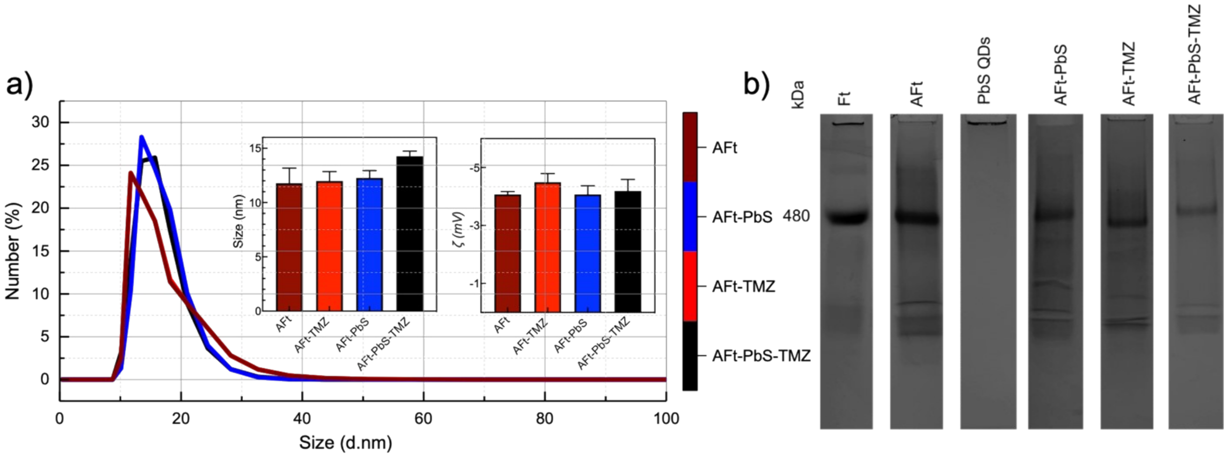

Figure S3. Synthesis and characterization of AFt nanoparticles. Size distribution and (insets) hydrodynamic size and zeta potential of AFt, AFt-PbS, AFt-TMZ, and AFt- PbS-TMZ measured by dynamic light scattering (DLS. Data are presented as mean ± SD of samples from three independent experiments (n = 3, N = 6). **(a)**, Native-PAGE gel results of horse spleen ferritin (Ft), horse spleen AFt, PbS QDs, AFt-PbS, AFt-TMZ and AFt-PbS-TMZ **(b)**.

TMZ release studies from AFt-TMZ and AFt-PbS-TMZ were carried out on a Slide A-Lyzer MINI Dialysis Device (ThermoScientific) at 0.1 M sodium acetate (NaOAc) buffer (pH 5.5) and 1X phosphate buffer saline (PBS) (pH 7.4). The formulations (n = 4, 400 μL) were placed in dialysis device and stored at 37 °C in NaOAc buffer or PBS. UV-Vis spectrophotometer was used to measure the release of nanoparticles at various time intervals (1h, 3h, 5h, 7h, 24h, 48h and 72h). The percentage of TMZ release was determined using the TMZ calibration curve (see Figure S1b). The following equation was used:

$$Drug\ Release\ \% = \frac{Amount\ of\ drug\ release\ (moles)}{Initial\ amount\ of\ drug\ (moles)} \times 100\%$$

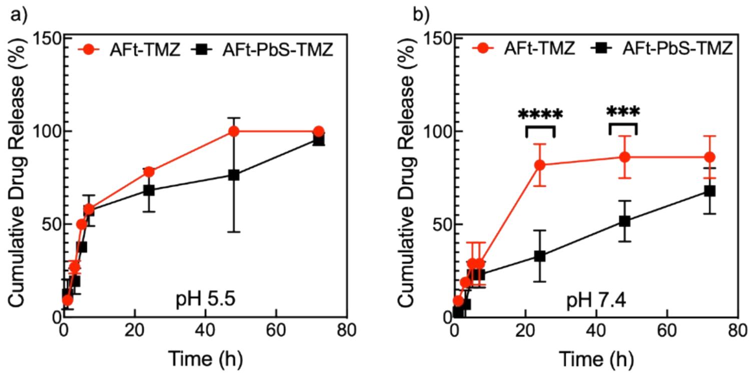

Figure S4. The comparable TMZ release profiles of AFt-TMZ and AFt-PbS-TMZ at pH 5.5 (a) and pH 7.4 (b).

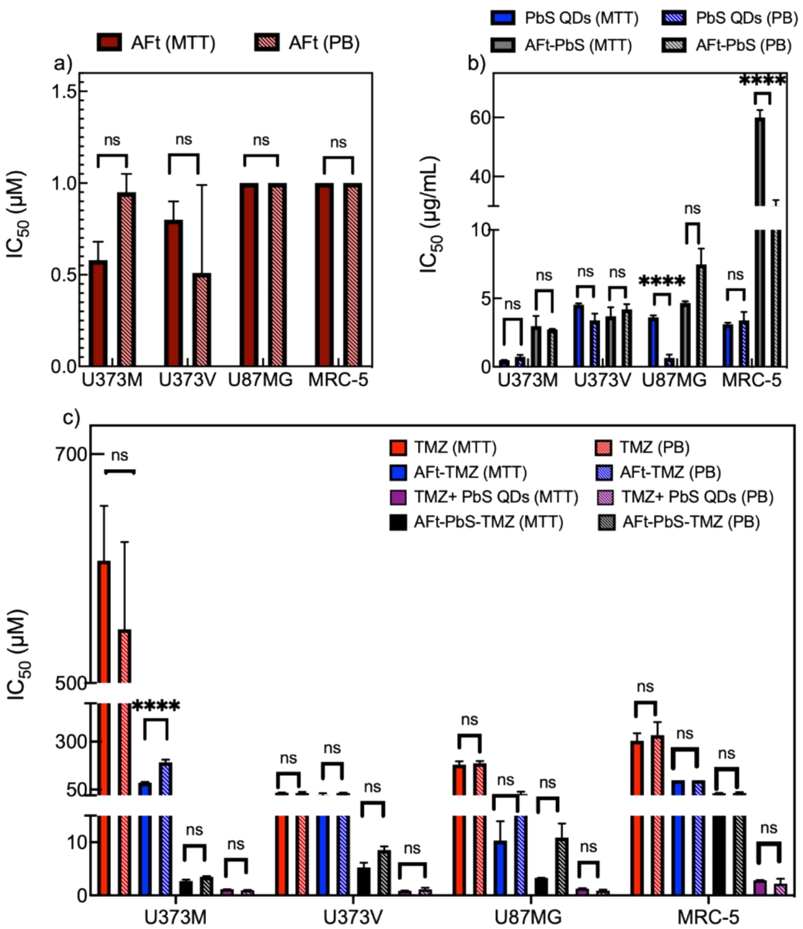

Figure S5. 2D comparison of naked and loaded formulations on cell proliferation by MTT and PB assays on GBM cell lines (U373M, U373V, and U87MG) and a healthy cell line (MRC-5). IC_{50} values comparison of AFt **(a)**, PbS QDs, AFt-PbS (**b)**, TMZ, AFt-TMZ, TMZ+ PbS QDs and AFt-PbS-TMZ **(c)** on GBM and MRC-5 cell lines. Data are presented as mean ± SD of samples from three independent experiments. (*n* = 3, *N* = 6).

The comparison of PB IC_{50} values across all examined cell lines following treatment with vehicle control, as well as both naked and loaded formulations. IC_{50} values following treatment of cells with AFt as a vehicle control revealed no significant difference in results between the MTT and PB assays, suggesting that AFt treatment had no effect on all studied cell lines (SI, Figure S5a). The overview of IC_{50} values after 6 days of PbS QDs and AFt-PbS treatment shows some differences in both treatments (SI, Figure S5b). Differences in sensitivity of, and results obtained from MTT vs PB assays generally may originate from treatment effects on metabolic activity. In terms of metabolic reactions, MTT measures the reduction of MTT dye by mitochondrial dehydrogenase enzymes in viable cells, resulting in the formation of formazan crystals. Conversely, PB assesses the reduction of resazurin to resorufin in viable cells, involving both mitochondrial and cytoplasmic enzymes. It is associated with cellular redox activity involving NAD(P)H.

In addition, IC_{50} values calculated for TMZ were as follows 610 ± 50, 34 ± 1 µM, 180 ± 20, and 310 ± 40 µM in MTT assay and 550 ± 80 µM, 33 ± 5 µM, 190 ± 10 µM, and 330 ± 70 µM in PB assay for U373M, U373V, U87MG and MRC-5 cells, respectively (SI, Figure S5c). The differences between IC_{50} values obtained for TMZ in MTT and PB assays were not statistically significant. However, evaluating cellular viability across diverse cell lines using these assays, through distinct metabolic pathways leading to the production of formazan and resorufin, brings attention to unique potential variations to each cell line. This variability can be related to cellular metabolism heterogeneity, enzyme expression and redox conditions among different cell lines. TMZ IC_{50} values on U87MG cells were reported to be 105 µM (five days treatment) and 300 µM (five days treatment) in several investigations [S1, S2]. These variations in IC_{50} values could be attributed to the use of various experimental techniques and cytotoxic tests. IC_{50} value for AFt-TMZ in U373M was considerably lower in the MTT assay than in the PB assay ($p<0.0005$). However, they have comparable IC_{50} values in other cell lines investigated. Lastly, in the co-encapsulated formulation (AFt-PbS-TMZ), no significant difference was observed across all studied cell lines (U373M: $IC_{50}<4$ µM, U373V: $IC_{50}<$ 9 µM, U87MG: $IC_{50}<12$ µM, MRC-5: $IC_{50}<38$ µM, human astrocytes: $IC_{50}<35$ µM). All data from MTT and PB assays indicate a congruence between the two assays. The discrepancies observed between the two assays may be attributed to their distinct working mechanisms. Boncler *et al.* conducted a comparison between MTT and PB assays on human endothelial cells treated with plant extracts, revealing variations in half maximum effective concentration (EC_{50}) values [S3]. Nevertheless, the PB assay is a good alternative for assessing cell viability in comparison to the traditional MTT assay and can be used to assess viability in 3D as well as 2D. This data suggests that the PB assay can be reliably employed in subsequent studies, including 3D tumour spheroids studies for evaluating 3D cell viability.

Table S1. IC_{50}(PbS QDs) obtained from PB assay performed in 2D and 3D U87MG cultures.

	**IC_{50} (PbS QDs) (µg/mL)**	
**Cell growth condition**	**PbS QDs**	**AFt-PbS**
2D	0.7 ± 0.3	7.5 ± 1.2
3D	26 ± 5	8 ± 1

Table S2. IC_{50}(TMZ) obtained from PB assay performed in 2D and 3D U87MG cultures.

		**IC_{50} (TMZ) (µM)**		
**Cell growth condition**	**TMZ**	**AFt-TMZ**	**TMZ+ PbS QDs**	**AFt-PbS-TMZ**
2D	190 ± 10	28 ± 11	1.6 ± 0.3	11 ± 3
3D	260 ± 30	180 ± 30	90 ± 6	5 ± 1

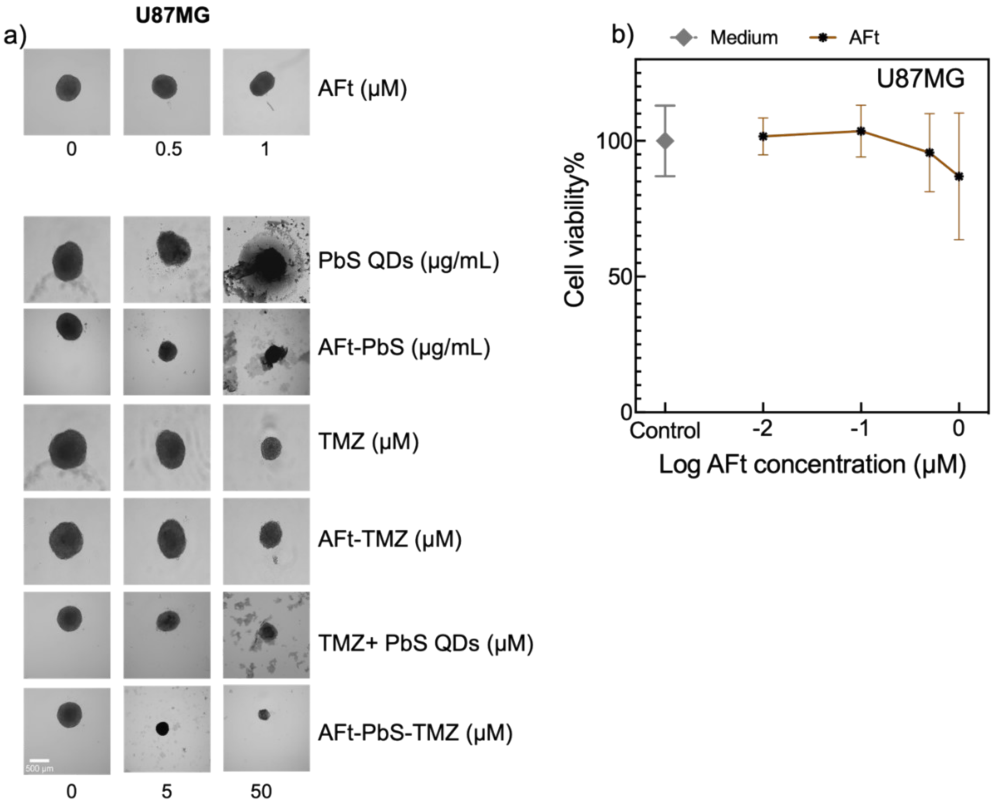

Figure S6. The effect of all formulations on 3D U87MG spheroids. Representative microscopic images of 3D U87MG spheroids on day 7 including control, 5 (µg/mL or µM) and 50 (µg/mL or µM) treatment (scale bar is 500 µm) **(a)**. The effect of AFt (vehicle control) on 3D U87MG cell viability with PB assay (**b**).

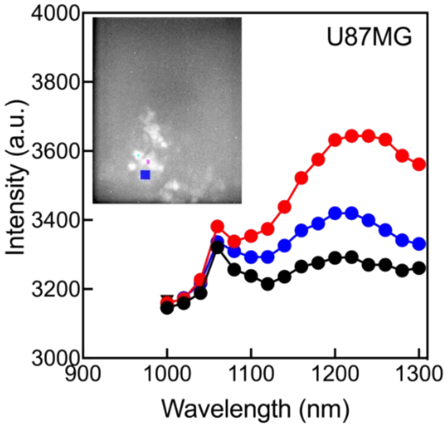

Figure S7. SWIR microscopy analysis of U87MG spheroids treated with 50 µg/mL AFt-PbS for 6 days using Z-sectioning. The inset shows a representative SWIR image of spheroid acquired at 20X objective. (785 nm laser, 980 nm LP, exposure time: 2 sec). The centre of the spheroids was selected for quantitative analysis. Intensity profiles from three selected areas presented.

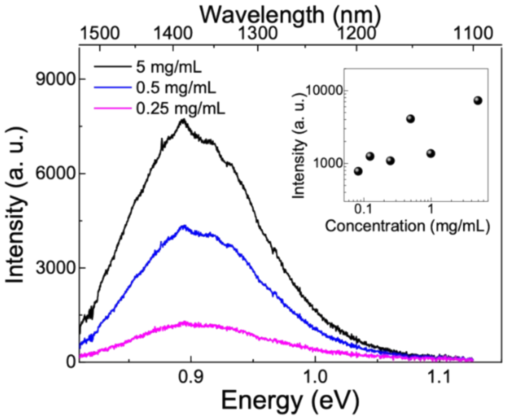

Figure S8. Photoluminescence of PbS QDs in deionized water with triethylamine at pH 11 (5 mg/mL, 0.5 mg/mL, and 0.25 mg/mL).

[S1] M. S. Lam, J. J. Aw, D. Tan, R. Vijayakumar, H. Y. G. Lim, S. Yada, Q. Y. Pang, N. Barker, C. Tang and B. T. Ang, Unveiling the Influence of Tumor Microenvironment and Spatial Heterogeneity on Temozolomide Resistance in Glioblastoma Using an Advanced Human In Vitro Model of the Blood-Brain Barrier and Glioblastoma. *Small*, 2023. p. 2302280.

[S2] V. Soni, M. Adhikari, H. Simonyan, L. Lin, J. H. Sherman, C. N. Young and M. Keidar,In vitro and in vivo enhancement of temozolomide effect in human glioblastoma by non-invasive application of cold atmospheric plasma. *Cancers*, 2021. **13**: p. 4485.

[S3] M. Boncler, M. Różalski, U. Krajewska, A. Podsędek and C. Watala, Comparison of PrestoBlue and MTT assays of cellular viability in the assessment of anti-proliferative effects of plant extracts on human endothelial cells. *J. Pharmacol. Toxicol. Methods***.** 2014. **69**(1):9-16.

ORIGINAL IMAGES OF THE DATE USED IN FIGURES

Figure 3b raw images: Original, uncropped, and unadjusted images supporting native-PAGE gel results.

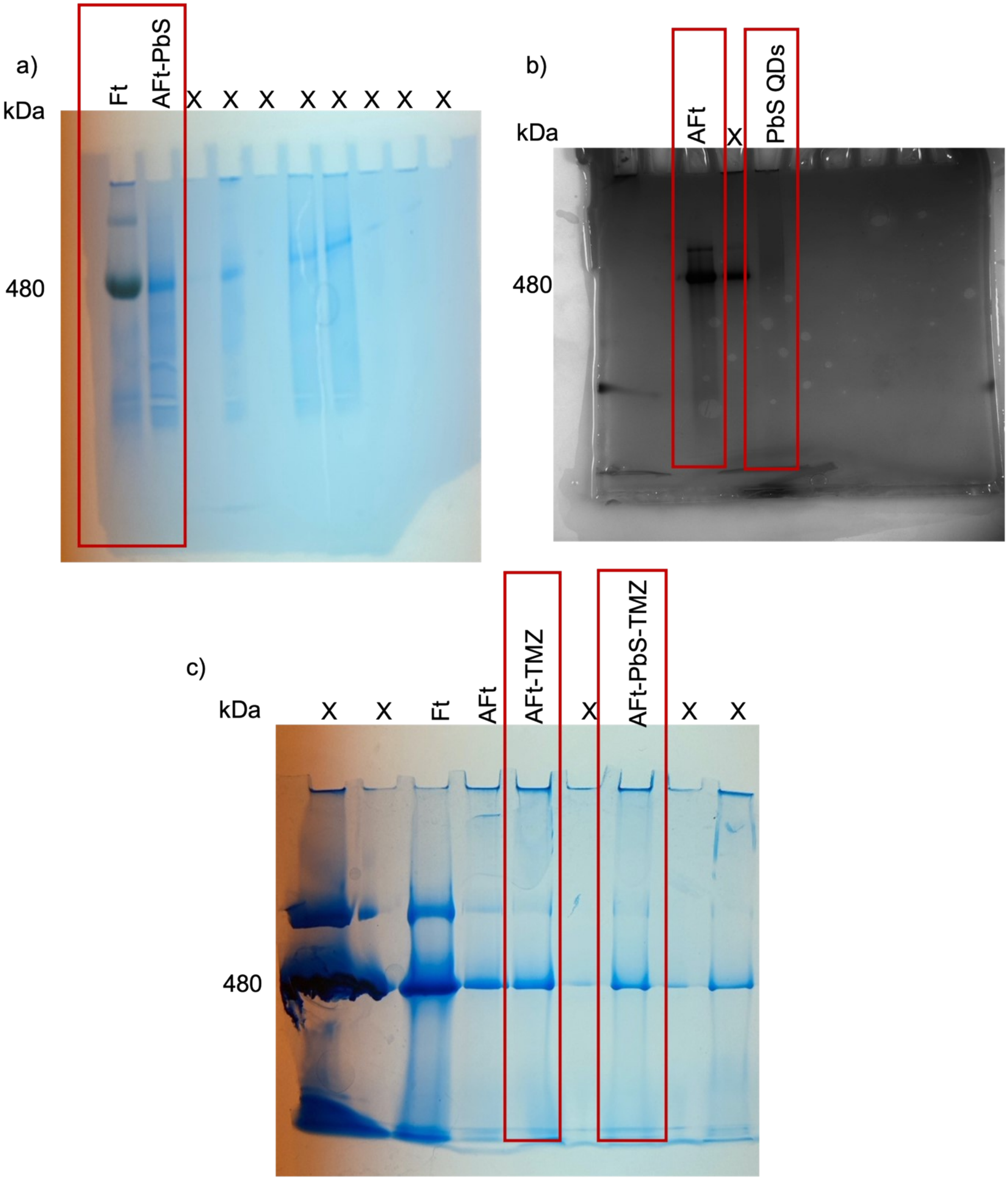

Figure S3b- raw image 1. Raw native-PAGE whole gel images of (a) Ft and AFt-PbS, (b) AFt and PbS QDs, (c) AFt-TMZ, AFt-PbS-TMZ shown in Figure S3b. Ft and /or AFt were used as a marker in each gel.

Figure 3a raw images: Original, uncropped, and adjusted images supporting western blot results.

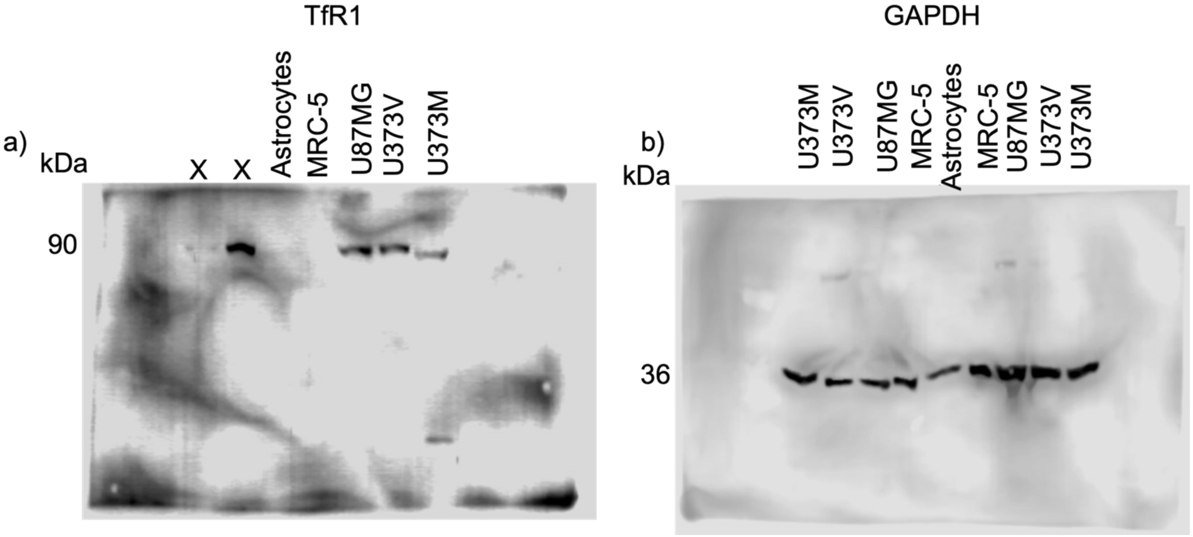

Figure 3a-raw image 1. (a) Uncropped western blot showing Tfr1 expression in astrocytes, MRC-5, U87MG, U373V, U373M cell lines. Data cropped, flipped, and used in Figure 3. Image was captured on LI-COR Biosciences after 5 min incubation with ECL substrate. (b) Uncropped western blot showing Tfr1 expression in astrocytes, MRC-5, U87MG, U373V, U373M cell lines. Data cropped and used in Figure 3. Image was captured on LI-COR Biosciences after 5 min incubation with ECL substrate.

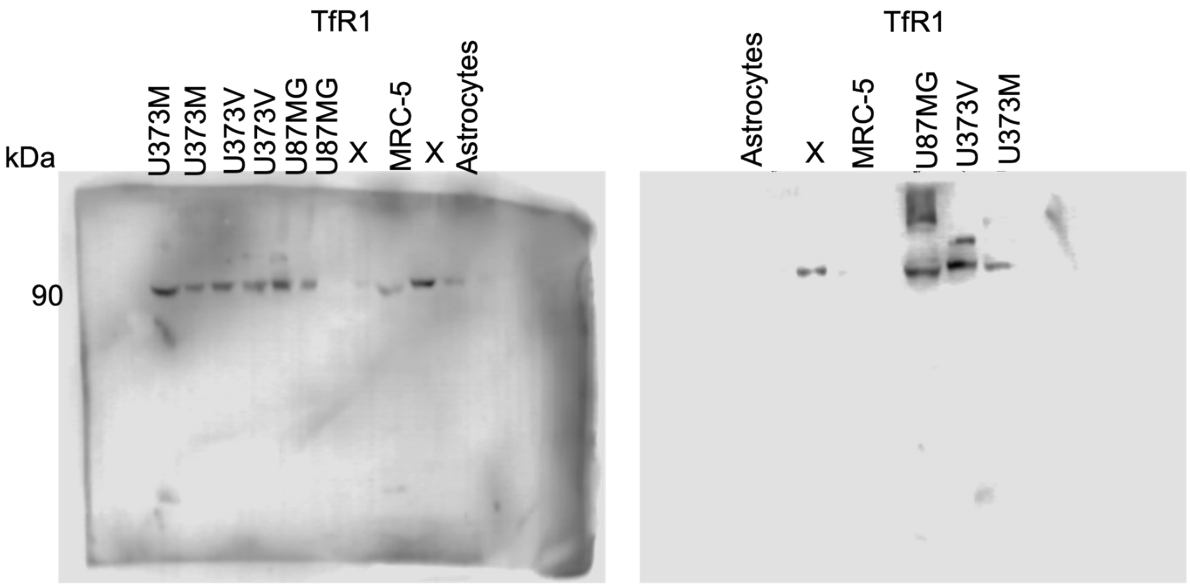

Figure 3a-raw-image 2. Uncropped western blot showing Tfr1 expression in astrocytes, MRC-5, U87MG, U373V, U373M cell lines (biological replicates). Images were captured on LI-COR Biosciences after 5 min incubation with ECL substrate.

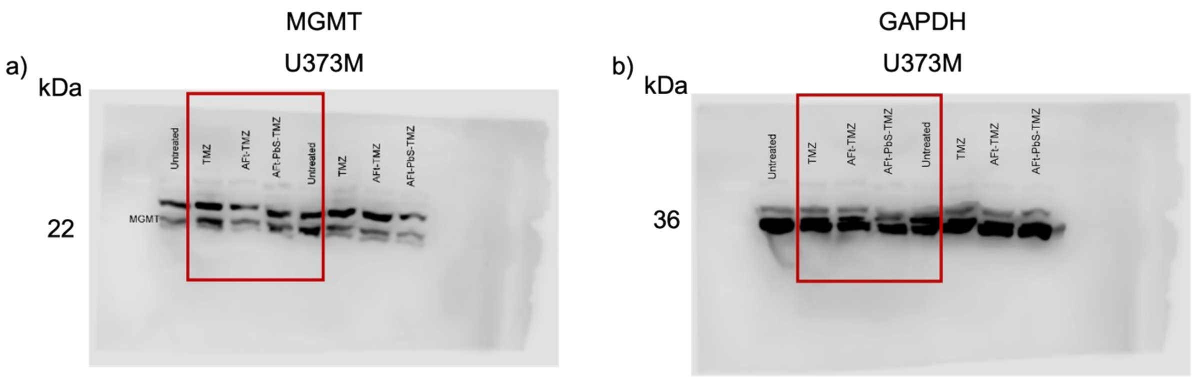

Figure 3b-raw image. (a) MGMT and (b) GAPDH expression by whole western blot image of untreated U373M (MGMT-positive) cells and following 6 days treatment with 10 µM TMZ, AFt-TMZ, and AFt-PbS-TMZ. Data cropped, flipped, and used in Figure 3b. Image was captured on LI-COR Biosciences after 5 min incubation with ECL substrate.
